# Factors influencing risk perception during Public Health Emergencies of International Concern (PHEIC): a scoping review

**DOI:** 10.1186/s12889-024-18832-z

**Published:** 2024-05-22

**Authors:** Jason Dean-Chen Yin, Juliana Nga-Man Lui

**Affiliations:** 1 Li Ka Shing, Faculty of Medicine, School of Public Health, The University of Hong Kong, 8 Sassoon Road, Pokfulam, Hong Kong China; 2 Faculty of Medicine, Department of Medicine & Therapeutics, Lui Che Woo Clinical Sciences Building, The Chinese University of Hong Kong, Prince of Wales Hospital, 9/F, Shatin, Hong Kong China

**Keywords:** Risk perception, Pandemics, Scoping review, PHEIC

## Abstract

The unknownness and dread potential of a risk event shapes its perceived risk. A public health emergency of international concern (PHEIC) declaration by the World Health Organisation (WHO) is a signal for such an event. Understanding perceived risk then shapes risk-avoiding behaviours, important for health prevention. The review aims to consolidate the determinants of risk perception during a PHEIC, underscoring the need for grounding in context and theory. Studies published from 2010 until end-2020, searching PubMed, PsycINFO, MedlinePlus, PubPsych, and CINAHL, were included. Studies with only biological conceptualisations of risk, or no association to risk perception, were excluded. A total of 65 studies were included. Quality of the cross-sectional studies was assessed using Newcastle Ottawa Scale (NOS), yielding an average of 5.4 stars (out of 10). Factors were classified into three broad categories – individual, contextual, and media. Individual risk factors include emotions; beliefs, trust, and perceptions; immutable physical traits (sex, age, ethnicity); mutable traits (education, income, etc*.*); and knowledge, with no definitive correlation to risk perception. Contextual traits include pandemic experience, time, and location, with only time negatively correlated to risk perception. Media traits include exposure, attention, and framing of media, with no clear association to risk perception. One limitation is excluding a portion of COVID-19 studies due to censoring. Still, this lack of consensus highlights the need to better conceptualise “risk perception”. Specifying the context and timing is also important since jurisdictions experience different outbreaks depending on outbreak histories. Using theories to ground risk perception research assists with these tasks.

## Introduction

A Public Health Emergency of International Concern (PHEIC) is defined by the World Health Organisation (WHO) as an “extraordinary event constituting a public health risk to States through the international spread of disease, potentially requiring a coordinated international response.” [1].The declaration of a PHEIC by WHO draws international attention, engendering a response by governments, media, and publics. Declaring the PHEIC serves as a signal to the world of an impending epidemic. Governments respond by implementing public health management strategies at a broad level (*e.g.* quarantines, travel restrictions, and public health education and communication) to steer individuals towards practicing preventive behaviours relevant to said pathogen (*e.g.* handwashing, physical distancing, mask-wearing, condom-wearing, etc*.*). The practicing of these preventive behaviours, if not mandated by governments, depends on one’s willingness to adhere, which depends on the level of risk a person perceives in relation to the pathogen. Understanding how risk perception is formed, and what factors shape it, is therefore important for preventing or slowing down transmission chains.

Before discussing risk perception, two issues must be reviewed. First, an overview of the conceptualisations of “risk” and “risk perception” is done. Second, this discussion extends into the realm of epidemics, introducing the topic of discussion: epidemic risk perception. Introducing these two issues lays the foundation for the current scoping review.

## Background: risk and risk perception

Studying risk requires an operationalisation for “risk”. This is difficult, since the pluralisation of risk studies in different fields (*e.g.,* economics, engineering, philosophy, health) narrows the definition depending on the aspect of risk in which the researcher focuses. To work around this pluralisation, we distil common elements of risk irrespective of field for a working definition. Risk can be distilled into having three elements: (1) a choice of action; (2) a probability of the risk event existing or occurring; and (3) a magnitude or consequences associated to the outcomes [2]. Explicit in this definition is that risk eventually involves *choosing* a course of action among many. Before this action is done, the process of choosing assumes the weighing of the possibilities of the risk event occurring, and the magnitude of consequences for a course of action. Implicit in this definition is that risk is all about behaviours, for if no action is done, no risk need be assessed. Early operationalisations of risk use the multiplication of (2) and (3) above to get a statistical calculation of risk [3].

Risk perception takes the concept of risk and expands it by focusing on processing. With momentum in from engineering and economics studies in the field of risk, researchers originally thought humans to be cognitive, rational decision makers. However, with the popularisation of the psychological approach in the mid-1960s, the field of risk perception expanded beyond the cognitive, rational individual to one of an emotional, contextualised, and irrational, faulty being. Early work by Slovic identified the importance of affect, emotion, and stigma in influencing risk perception, laying groundwork for studying the emotional dimension of risk perception that complemented the cognitive [4]. Douglas proposed the Cultural Theory of Risk that accounted for the context and experiences of individuals in societies. Her theory stipulated that risk perception reflects the underlying collective and shared conventions of a society [5]. Kahneman and Tversky’s work on event probability evaluation led to the studies of heuristics and biases – cognitive shortcuts and mishaps, respectively – that prevent humans from “thinking clearly” [6]. The incorporation of the affective, the contextual, and the irrational thinker expanded the risk perception field, deviating it from a purely technical, acontextual process. The implication for risk studies is that it is ultimately subjective. Risk perception discards the notion of a universal risk by allowing for variation in the way people recognise, perceive, and process the world, and the outcomes they wish upon themselves and others [7]. Risk perception is thus defined as the subjective judgment(s) about the severity of a risk that account for the experiences of individuals in different contexts.

With an understanding on the definitions of risk and risk perception, the next section explores this relation in the outbreak context.

### Outbreak risk perception

Slovic et al. classified a selection of risk events through a bi-axial taxonomic system [8]. Each risk event was boiled down to vary along the intersection of two dimensions – *unknownness* and *dread* – and was factorially mapped in a 2-D plane. Unknown risks were phenomena which were perceived as novel, non-observable, new to science, and with delayed effects. Dreaded risks would be those defined by having involuntary, uncontrollable, fatal/catastrophic consequences. The range of risk events chosen, and their mappings highlighted a major point in studying risk perception: the risk event itself matters for risk perception.

A PHEIC is likely to rank high on both *unknown* and *dread* dimensions. Part of this is inherent to the signalling mechanism of a PHEIC; that the leading, international health body (WHO) has deemed the outbreak a growing global threat sounds alarming. This alarm, coupled with an initial information gap about the origin or spreading potential of a newly emerging, or re-emergent, virus; the infectious process being invisible to the naked eye; and the delay of the potential impact make it a highly unknown risk. In addition, the (presumed) fatality of infection; the fear of contagion; the potential global impact; and the initial lack of a foreseeable cure all point in the direction of high dread. Although some heuristics and past pandemic experience may mitigate the unknown or dread potential, it is unlikely to happen at a level that would quell increased perceived riskiness as the disease spreads.

During a PHEIC, adhering to behaviours that experts recommend is important. Under the assumption that people want to avoid illness, researchers have examined upstream factors predicting behaviours. Again, the current discussion emphasises a focus of risk studies on behaviour. Two major theories in health psychology elaborate on the importance of risk perception to behaviour: the Health Belief Model (HBM) [9, 10] and Protection Motivation Theory (PMT) [11]. These two, although slightly nuanced, have four overlapping conceptualisations of risk perception that are determinants of health behaviour: (1) *perceived susceptibility*, which is the beliefs about the susceptibility to the disease; (2) *perceived severity*, the beliefs about the seriousness of the health risk and adverse consequences; (3) *perceived benefits*, the beliefs about whether a health behaviour helps manage risk; and (4) *perceived barriers*, the belief on the costs of adopting a health behaviour (including the conceptualisation of *self-efficacy* – the ability to carry out the behaviour – and *response efficacy –* the efficacy of the behaviour to avert the threat itself – as noted in PMT*)*. Important to note here is that the first two metrics – susceptibility and severity – refer to the disease itself, whereby the latter two – benefits and efficacy – focus on the response to the disease. To understand any behaviour, both the perception of the disease and the avoidance method(s) is important and will be discussed later.

**Fig. 1:**
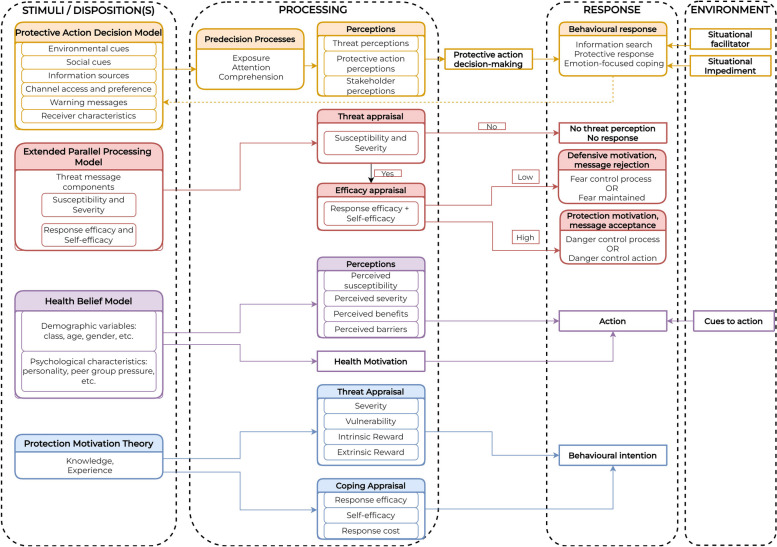
Consolidation of concepts in frequently used health behaviour theories

Theories linking risk perception to behaviour also exist in other fields. Several other theories focus on the communication of messages and have features of risk perception and behaviour. One such example is the Protective Action Decision Model (PADM), focusing on how threat perception (*i.e.* perceived severity and susceptibility) and protective action perceptions (*i.e.* perceived barriers, self-efficacy) influence protective action decision making in the event of a hazard (*i.e.* a risk event, or in this case, a PHEIC) [12]. Another is the Extended Parallel Processing Model (EPPM) [13, 14] that look at how individuals respond to fear-appeal (*i.e.* fear-inducing) messages. The EPPM also has constructs of perceived threat and perceived efficacy which mirror that of PADM, HBM, and Theory of Planned Behaviour (TPB) (in fact, EPPM was influenced by TPB). An overlap of HBM, TPB, PADM, and EPPM are done in Fig. 1 to summarise conceptual similarities and differences in the approach to risk perception research for health.

Three things must be noted from this figure. First is the focus of all theories on explaining behaviour. All theories head towards the direction of a response. Second is the central component of risk perception to behaviour; that before a decision is made, some processing – whether cognitive or reactive (emotional) – must occur. Third, there are a host of upstream factors that may affect the risk processing and perception. These three are important considerations for the present review.

Two previous reviews studied risk perception in relation to pandemics. The first by Leppin and Aro concerns two thematic areas: the conceptual and operational definitions of “risk” during Severe Acute Respiratory Syndrome (SARS) and the 2009 pandemic flu, as well as the relationship of risk perception to behaviour [15]. On concepts and measurements, the review revealed the heterogeneity in conceptualising pandemic risk due to the pervasive ‘lack of conceptual elaboration’ on the perceived risk concept within a study. Most of the concepts were inferred through the operational tool, *i.e.*, the measurement instruments. Using this indirect method, they elucidated five thematic concepts of risk: (1) the object or the ‘who’ of risk perception (self, country, society, world); (2) a comparison against other disease risks (pandemic versus other health or social risks); (3) situational risk, or the ‘when’ of risk (in a hospital, travelling on a plane); (4) the health-disease spectrum of risk (contracting virus versus death); and (5) risk acceptance (how much the risk was acceptable). Multiple conceptualisations of risk perception makes consensus-building around risk perception difficult since measurements and comparisons are across different concepts of risk.

The second part of Leppin and Aro highlighted different models that link risk perception to behaviour. There is a lack of studies that study the complexity of the decision-making process on behaviours. This is corroborated by the predominant use of correlational studies which disallows a true study on behaviour motivation *due to* certain risk perceptions. Rather, what is being studied is a measurement of fit between perceptions of own protective behaviours and risk – what Leppin and Aro call an *accuracy hypothesis* [16] – since the performance of protective behaviour likely also means a resulting lower risk perception. This finding underlines another important component to risk perception research: time-sensitivity. At the initial declaration of a PHEIC, where the threat is novel, the issue of perception-behaviour directionality may be less important since there has been less time to practice preventive behaviours. Studying this short window reduces the concern on internal validity since it is a hazard that has not operated on a long timeframe (*i.e.* such as for chronic diseases, where perceptions and behaviours constantly change). This scenario references the high dread nature proposed in Slovic’s two-dimensional framework. In this case, a longitudinal study looking at the risk perception-behaviour relationship at the initial outbreak would capture the link between risk perception and behaviours more accurately. Indeed, in one study of SARS in 2003, one study revealed a steady increase then levelling off in risk perception in later phases [17]. Both the concepts of risk perception and the importance of time will be extracted from the studies in this review.

The second review elaborates on the relationship of risk perception and behaviours during outbreaks, especially with regards to knowledge, awareness, and misconceptions [18]. To do this, authors focused on five major “pandemics or outbreaks” in the twenty-first century: SARS, pandemic flu of 2019, Middle East Respiratory Syndrome (MERS), Ebola, and Coronavirus Disease of 2019 (COVID-19). In their cataloguing of information sources, they found that most participants relied on multiple information sources – social media, print media, government websites, family members – from both local, regional, and international levels to inform them about pandemic knowledge. Another category of findings was that on misconceptions; misconceptions about the infection; misconceptions on what treatments worked; doubts as to the origin of the outbreak agent; as well as spreading of misconceptions. While their finding was that knowledge was linked to risk perceptions and behaviour, this conclusion may have been drawn from the *accuracy hypothesis* style of reasoning mentioned above. The important point from this review – in addition to Leppin and Aro’s – is that information and its communication in the first stage of an outbreak is important in how it influences risk perception.

While these two studies lay groundwork for studies on risk perception during outbreaks, each miss what the other has. Leppin and Aro have not explored what upstream factors shape risk perceptions, despite their conceptual and operationalisation clarification. Their classification schema, however, is a useful tool for consensus building if used as a conceptual map. The study by Majid et al*.* alludes to upstream factors in their exploration of information but does not consolidate findings against multitude conceptualisations of risk perception.

There are also blind spots in both reviews. Neither accounts for context nor the different resulting outcomes from context. Context may manifest in different ways; in different populations, different countries and their political trajectories, different pandemics, or even in the timing of a study within a the same context. In addition, there is no exploration as to the types of frameworks or theories used in risk perception research. These are useful to elucidate how researchers approach risk perception studies; essential to establish consensus in the field. The motivation of this review is to thus do several things: (1) consolidate the factors that shape risk perception, mapping them to the different concepts of risk perception; (2) understand how differing contexts may yield different findings; and (3) identify any theoretical approaches to risk perception studies to suggest a way moving forward.

## Methods

Under the Population, Concept, Context (PCC) framework suggested by the Joanna Briggs Institute [19], we aim to keep the scope broad by including a breadth of global studies to ensure that a diversity in experiences of risk perception – however conceptualised – is included. For population, studies will not be limited by region, age, gender, education, or any other demographic factor as it aims to capture risk perception across all experiences. The acquired studies may be assorted *ex-post* but will not be a factor for study selection. Any studies focusing on one specific group of people (*e.g.,* health care workers, nurses, multiple chronically ill persons) will also be included. Since this study focuses on risk perception *during a PHEIC*, any studies including risk and/or risk perception or related terms will be included, regardless of the type of study carried out (*e.g.,* observational, cohort, etc.). In addition, all studies must at least identify, and measure risk perception (or a related concepts, *e.g*., vulnerability, susceptibility, severity) from the data collection tool *and* have associative analysis done to find its predictors. This excludes studies that use risk perception exclusively as a predictor for another outcome but includes those in which risk perception is either the main outcome, or mediator in analysis. In addition, since the study focuses on risk perception and not absolute risk, nor epidemiological risk, any studies with an outcome of solely a biological or epidemiological risk outcome (deaths, hospitalisations, cases, etc*.)* are excluded. In a preliminary search, many studies relate to risk perception of vaccines for the specified PHEIC. Although this is an important anti-epidemic behaviour of pandemic control, these types of studies are excluded because the present study focuses on the importance of the unknownness and dread of PHEICs (*i.e.,* in the initial stages of an outbreak prior to vaccination), assuming that at time of release of vaccination, the PHEIC would have already entered a later stage. There will be no limitations as to the context, location, or timing of the study to capture a global and temporal scope on the variety in risk perception research.

Studies will include those ranging from 2003–2020 (inclusive) to include recent PHEICs; the two sandwiching years indicating the 2003 SARS outbreak and COVID-19 outbreak years. The end-2020 censorship is to both remove the presumed over-representation of COVID-19 studies, as well as reduce the inclusion of vaccine risk perception-related research for COVID-19. SARS was included even though it predates the PHEIC concept since it is closely linked to the development of the concept and was an early epidemic in recent consciousness of which to have shaped the IHR revision and PHEIC conceptual development. Other notable outbreaks of international concern that have existed for several decades (HIV) or centuries (Dengue, etc*.)* are not included because a lack of a formalised ‘warning mechanism’ to signal a growing threat, as well as their long durations which confound the question around time. The review will be limited to studies done in English and includes those that are peer-reviewed, and either published or in pre-print. Grey literature was searched through Google Scholar to identify any other types of documents that would study risk perception during PHEICs. To do this, the same search terms were used, but domains were specified to “.org”, “.gov”, “.com” suffixes, and restricted to file types of pdfs, documents, or Powerpoints. In addition, OpenGrey from the European Union, and Wonder from the USCDC, were searched for corresponding studies.

The search strategy was as follows. We first discussed the different ways ‘risk perception’ could be asked to capture the potentially different concepts of risk perception. This included terms such as “perceived susceptibility”, “perceived vulnerability”, “perceived risk”, “worry”, “perceived severity”, as derived from the health behaviour literature. Afterwards, we collected the assortment of PHEICs that have occurred within the specified time frame, which includes SARS, H1N1, Ebola, Zika, COVID. Polio was not included due to it having a vaccine. In addition, other notable outbreaks of epidemic concern such as MERS were excluded given their exclusion as a PHEIC. Articles containing any variety of ‘risk perception’ and a PHEIC, within the specified date range (2003 to end 2020) and language restrictions, in the title and the abstract, were imported into Covidence to prepare for analysis. The generic search strategy is below:

(((“risk perception” OR “perceived susceptibility” OR “perceived vulnerability” OR “perceived risk” OR “worry” OR “perceived worry” OR “susceptibility” OR “vulnerability” OR “fear”) AND (SARS OR “pandemic influenza” OR avian OR H1N1 OR swine OR ebola OR zika OR COVID OR ncov OR coronavirus)) AND ((”2003/01/01”[Date - Create] : 2020/12/31”[Date - Create]) AND (english[Language])) NOT vaccine.

An overall search of five databases were done: PubMed, PsycINFO, MedlinePlus, PubPsych, and CINAHL. Lastly, the reference lists of all identified reports and articles were searched for their title and abstract to see if they also contained the terms as above. Once the list of sources was compiled, one further filtration step followed. The entire body of all pieces were screened briefly to see if studies explicitly mentioned risk perception as a measured variable, regardless of whether as an outcome, mediator, or predictor. This was done through looking for the keywords in the search criteria above separately so as to identify the concept of risk, and see whether it was measured. These steps were completed independently by both authors, and any discrepancies were resolved through discussion. After completing these steps, the data charting began.

A charting form was composed in Covidence and used to extract data from the remaining studies post-screening. The extraction could be categorised into three broad sections. The first section contains study characteristics that may differentiate the studies among each other, including: the title; type of study done; country; pandemic studied; specific subpopulation (*e.g.,* doctors, taxi drivers); number of participants. The second section contains results on the theoretical conceptualisations to risk perception studies; the answer to *what* is risk perception. This section includes: any specific theories invoked (*e.g.,* Health Belief Model, Social Amplification of Risk); the different categories of questionnaire items asked; and the concept of risk assessed. For the concept of risk assessed, we used a collapsed version of Leppin & Aro classification schema from their 2009 paper [15] and included five classifications: (1) object of risk perception (who the risk is for); (2) risk comparison against other disease risks; (3) situational risks (*e.g.,* on a plane, in a hospital); (4) health-disease continuum (*e.g.,* getting infected, falling ill, or dying); and (5) risk acceptance. The third section contains results on *where* in the pathways risk perception is important in PHEICs, *how* it is shaped, as well as *when* the studies are important. This section includes where risk perception lies in the pathway (mediator, outcome); identifying what factors the author found to associate with risk perception; and study dates to highlight the importance of temporality in risk perception studies. The results from the third section will map onto the different conceptualisations of risk deduced from section two to consolidate findings. For quality checks of the studies, the Newcastle Ottawa Scale adapted for cross-sectional and longitudinal data was used. Both authors independently conducted the charting process and discussed any discrepancies.

As many studies focused on measuring risk perception, one further screening step was added later and applied. For studies that do not measure any *association* to risk perception, they are not included. Since the purpose of this scoping review is to find factors associated to risk perception, those that simply measure it without formation or association to it are further excluded. In accordance with the scoping review protocol proposed by the Joanna Briggs Institute [19], the summary of the data was done in both tables and charts to summarise how the studies varied in accordance to location, population, time, and concept of risk perception. Subsequently, the authors expound in narrative format to flesh out the complexities of the selected risk perception studies. This study has been registered in PROSPERO under ID number CRD42022349067.

## Results

### Search results

**Fig. 2:**
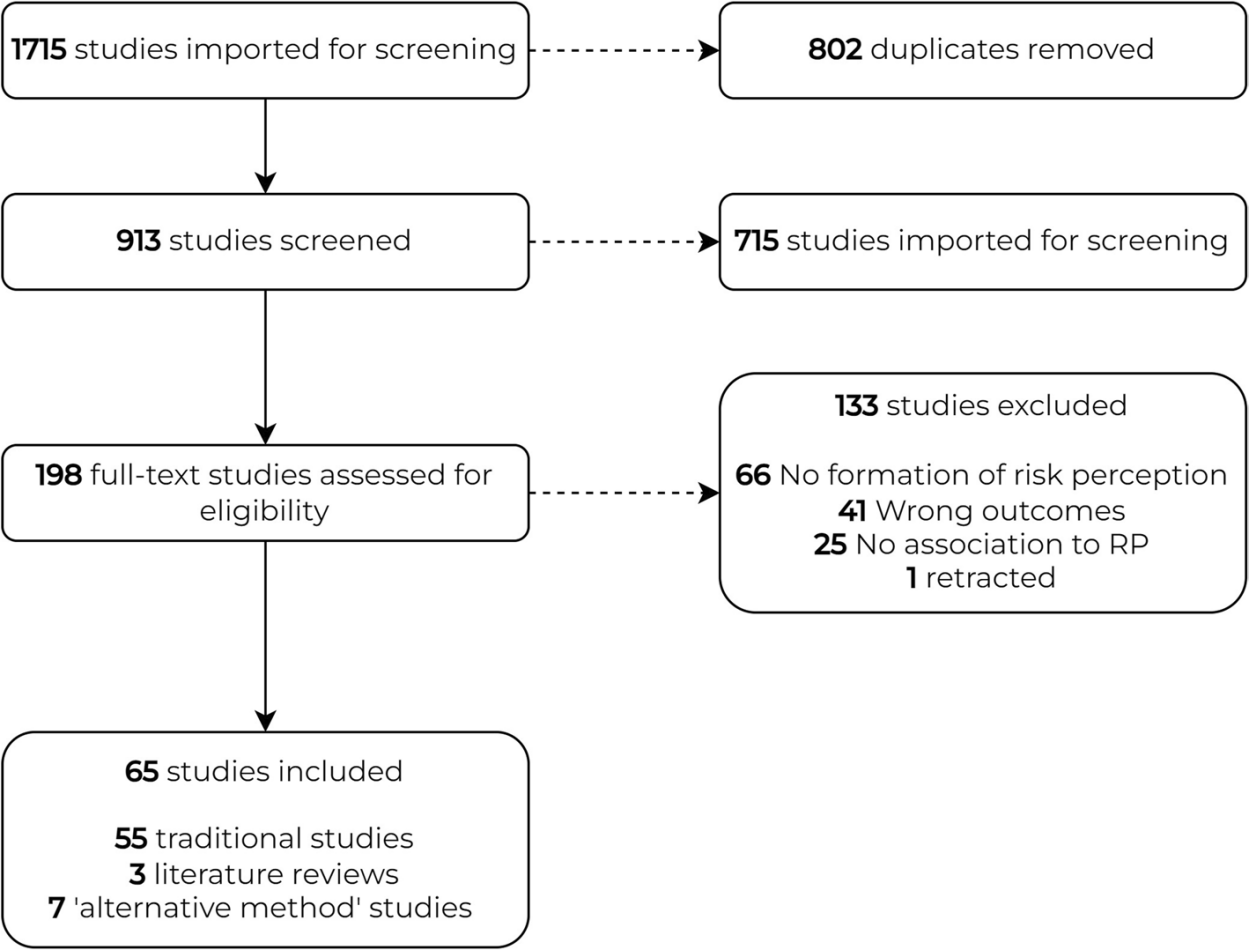
PRISMA flowchart

Initial systematic search of electronic databases identified a total of 913 studies. Of these, 715 were deemed irrelevant based on a screening of the abstract due to overall subject matter irrelevance (*e.g.* focus on non-PHEIC diseases such as HIV or other viral agents), leaving 198 studies for full-text analysis. A further 132 studies were excluded after full-text analysis for one of several reasons: there was no association towards risk perception formation; the study was a description of how to measure risk perception; the study mentioned “risk” and “pandemic” but was not about a pandemic risk. Overall, 65 studies were included as part of this systematic search. The full process for the search is presented on the PRISMA flow-chart below (Fig. 2).

### Summary characteristics

In total, 196 studies were eligible for inclusion. The included studies (*n* = 65) were categorised by study type, location, PHEIC, utilised theories, how risk perception was used as a variable in the study. Excluded studies (*n* = 133) that are not cited here were excluded on bases of not measuring risk perception per the specified inclusion criteria (*n* =66), an outcome unrelated to risk perception (*n* =41), no associative analysis to risk (*n* =25), and one retracted study (*n* =1).

Overall, 43 (66.2%) of included studies used a cross-sectional method; 6 (9.2%) used a repeated-cross sectional method, 2 (3.1%) used a longitudinal or cohort method; 3 (4.6%) were literature reviews; 7 (10.8%) were ‘alternative methods’; 3 (4.6%) were experiments; and 1 (1.5%) was a mixed method study. The ‘alternative studies’ is an overflow category containing a mixture of different analytical frameworks, analysis methods, and conceptualisations of risk perception. They are elaborated on in the discussion due to their contributions to expansion of the risk perception field. Literature reviews will be charted separately as well since they do not conform to the categories for charting. The following results do not include literature searches (*n* = 3) in the denominator.

By far, the most represented regions are Asia (*n* = 18, 29.0%), North America (*n* = 17, 27.4%) and Europe (*n* = 13, 21.9%). In Asia, most studies are about China (*n* = 8). North American studies are all represented by the United States (*n* = 17), and European studies are mostly about Italy (*n* = 5) or The Netherlands (*n* = 3). Few studies regard the African continent (*n* = 3, 4.8%), Middle East region (*n* = 2, 3.2%), and South American (*n* = 1, 1.6%). Several studies compared across continents (*n* = 6, 9.6%). One study did not explicitly mention a location, nor could it be extrapolated from the study text.

The most common PHEIC studied was COVID-19 (*n* = 41, 66.1%), then Ebola and H1N1 (*n* = 8, 12.9%), Zika (*n* = 4, 6.5%) and SARS (*n* = 2, 3.2%).

Theories were very sparsely used throughout the studies to guide analytical methods. Overall, 29 studies (46.7%) of studies used at least one theory. For some studies, multiple theories were used. The most popular two theories were the HBM and PMT (*n* = 7, 11.3%). These two theories are the dominant theories in health psychology and corroborate the findings on their common use in Leppin and Aro [15]. Other lesser used theories include TPB (*n* = 3, 4.8%), EPPM (*n* = 2, 3.2%), and PADM (*n* = 1, 1.6%). While some studies used at least one theory to guide their analytical approach (*n* = 13, 21.0%), over half did not have any theoretical approach (*n* = 38, 61.2%).

The current review only included studies that assessed an association with risk perception. Thirty-five included studies assessed risk perception as the main outcome (*n* = 34, 54.8%), and 25 (*n* = 40.3%) used it as a mediator for another outcome. Of those that used it as a mediator, risk perception was most often correlated with performing public health behaviours (*n* = 18, 72.0%). The other studies looked at a variety of different outcomes such as affective response (*n* = 2, 8.0%); economic confidence (*n* = 1, 4.0%); public acceptance (*n* = 1, 4.0%); depression (*n* = 1, 3.8%); or in-group bias (*n* = 1, 4.0%). Three studies, one each, looked at performing public health behaviours in addition to an outcome representing “support” (such as trust in information; support in institutions; or support for pandemic policies). These results are summarised in Tables 1, 2 and 3.

**Table 1:** Summary tables for epidemiology studies, literature reviews, and alternative studies assessing risk perception. Epidemiological papers assessing risk perception

**Author and Year**	**Study design/Country**	**PHEIC**	**Theories**^a^	**Sample and sampling method**	**Leppin-Aro Concept**^**b**^	**Factors associated with higher RP**^c^	**Factors associated with lower RP**	**Where RP on pathway**	**Study periods, n** **Special timing**	**NOS**^d^
Abir et al. 2020 [20]	Cross-sectional /Bangladesh	COVID-19		Adults	1, 4	More practise of self-quarantining Never quarantined before (mandatory); More worry	Later lockdown; Being female; Being non-Muslim;	Main outcome	Mar 26–31, 2020; (*n* = 322) Early lockdown May 11–16, 2020; (*n* = 683) Late lockdown	6
Alschuler et al. 2020 [21]	Cross-sectional/United States	COVID-19		Multiple Sclerosis patients, over 18 years old	1, 4	Having COVID-19 symptoms; Having COVID-19 risk factors Higher anxiety;	Being older; Having higher positive affect and well-being	Main outcome	Apr 4 – May 26, 2020 (*n* = 491)	6
Asefa et al. 2020 [22]	Cross-sectional/Ethiopia	COVID-19		Waiters	1, 2, 3, 4	Older; More knowledge of COVID-19; More preventive behaviours practiced		Main outcome	Jun 1 – 15, 2022 (*n* = 416)	7
Brug et al. 2004 [23]	Cross-sectional/Netherlands	SARS		Random internet sample, aged 19–78	1, 2, 4	Having less education Being female		Main outcome	Jun 19 – 26, 2003 (*n* = 373)	2
Bults et al. 2011 [24]	Repeated cross-sectional/Netherlands	H1N1	PMT; HBM	Adults, aged 18 and over, representative internet panel	1, 4		Time	Mediator; Outcome: taking preventive measures	Apr 30 – May 4, 2009 (*n* = 456) Jun 15–19, 2009 (*n* = 478) Aug 11–20, 2009 (*n* = 934)	7
Dai et al. 2020 [25]	Cross-sectional/China	COVID-19	Protective Action Decision Model	Recruited online, 33 provinces in China	4	More positive risk information; More detailed pandemic information		Mediator; Outcome: perform PHB	Feb 24 – Mar 3, 2020 (*n* = 1,131)	5
De Coninck, d’Haenens, Matthijs 2020 [26]	Cross-sectional/Belgium	COVID-19		Online survey, aged 18–70, Flanders	1, 4	Older age; Lower education; Being female; No telecommuting available for work		Mediator; Outcomes: (1) Public acceptance of measures; (2) Belief on gov’t handing of crisis	Mar 17 – 22, 2020 (*n* = 1,000) During first restrictive epidemic measures	7
de Zwart et al. 2009 [27]	Cross-sectional/Multiple (five European countries; three Asian regions)	SARS	PMT	Computer-assisted telephone interview, random digit dialling, aged 18 to 75	1, 2, 4	Being female; Lower education; Higher SARS knowledge		Main outcome	Sep 20 – Nov 22, 2005 (*n* = 3,436)	4
Ding et al. 2020 [28]	Cross-sectional/China	COVID-19		WeChat and QQ, College students and university professors in China	1, 4	Being female; Exposure to confirm/suspect cases; Higher knowledge; School location in Hubei		Main outcome	Feb 4 – 7, 2020 (*n* = 1,461) First outbreak in China	3
Dryhurst et al. 2020 [29]	Cross-sectional/Multiple countries (five European; two Asian; two North American; Australia)	COVID-19		Several different platforms (country-specific); quota sampling for some; administered through Qualtrics	1, 4	More direct personal experience with virus (third most important); More information received on virus from family and friends; More altruistic/prosocial (second most important); Higher belief in personal efficacy; Increased trust in science, medical practitioners; Higher personal knowledge	More individualistic (ranked most important); More trust in government; Being male	Main outcome	Mar to Mid-Apr 2020 (*n* = 6,991)	7
Duculan et al. 2020 [30]	Cross-sectional/United States	COVID-19		With systemic rheumatic disease; patient recruitment	1, 4	Having rheumatic condition; Taking certain rheumatic medications; Having higher medication necessity score; Higher anxiety and depressive symptoms; Lower physical function		Main outcome	Apr 2 – 21, 2020 (*n* = 112) Height of COVID-19 pandemic in New York City	6
Farooq, Laato, Islam 2020 [31]	Cross-setional/Finland	COVID-19	PMT	Webropol online survey; sent to students, faculty, employee	1, 4	Increased ‘cyberchondria’ – impulse to go online to find further reading on a health topic (info overload)		Mediator; Main outcome: self-isolation intention	Mar 19 – 30, 2020 (*n* = 225)	5
Frissen et al. 2020 [32]	Repeated cross-sectional/Belgium	COVID-19		Flanders, adults aged 18–70, through polling agency iVOX	1, 4	Having higher overall media exposure; Access to public/quality media;		Associated factor; Outcome: practising self-protective behaviours	Mar 17 – 22 (*n* = 1,000) Three days after restrictions were implemented Apr 6 – 18, 2020 (*n* = 870) Peak of outbreak in Belgium	7
Harapan et al. 2022 [33]	Cross-sectional/Indonesia	COVID-19		Community members in Indonesia through Google Forms	1, 4	Being older; Being unmarried; Having higher individual monthly income; Living in urban cities; Being a health care worker		Main outcome	March 25 – Apr 6 (*n* = 1,379) Recommended to stay at home	3
Han et al. 2014 [34]	Cross-sectional/United States and China	H1N1		Online survey to college (university) students	1, 4	Distance from disease; Increased interpersonal communications; Increased exposure to social network sites; More attention to H1N1 flu information	Higher self-efficacy	Main outcome	Summer 2010 (*n* = 2,987)	
He et al. 2020 [35]	Cross-sectional/China	COVID-19		Chongqing residents living in Chongqing within last 6 months, convenience sampling	1, 4	Being female; Being older; Higher household income; Living with children; Using TV, community workers, or free media websites as main source of information; Living with family members with chronic diseases	Those using WeChat contacts as main source of information	Main outcome	Feb 13–14, 2020 (*n* = 476) When residents returned to work	7
Hubner et al. 2020 [36]	Cross-sectional/United States	Zika	PRISM	Childbearing age Floridians (aged 20–40), recruited by Qualtrics; purposive sampling	1, 4	Being or planning to be pregnant		Mediator; Outcome: affective response; information seeking intention	December 2016 (*n* = 494)	5
Huynh 2020 [37]	Cross-sectional/Vietnam	COVID-19		Random sample of “internet research source”	4	Higher frequency use of social media; Being from central/southern Vietnam		Main outcome	Feb 1 – Feb 20, 2020 (*n* = 391) Vietnamese prime minister declares global and national emergency	6
Ibuka et al. 2010 [38]	Repeated cross-sectional/United States	H1N1	HBM	Survey firm, United States general sample	1, 4	Living in states with H1N1 incidence; Being female; Larger households		Mediator: Outcome: perform PHB	Apr 28 – Apr 30, 2009 Apr 30 – May 12, 2009 May 19 – May 26, 2009 (*n* = 1,290)	7
Iorfa et al. 2020	Cross-sectional/Nigeria	COVID-19	HBM	Self-adminsitered questionnaire, general population aged 15 and above, across 36 states of Nigeria	1, 4	Having more COVID-19 knowledge (for men); Being older		Mediator; Outcome: precautionary behaviour	Apr 20 – 30, 2020 (*n* = 1,554)	7
Johnson 2017 [39]	Cross-sectional/United States	Ebola		Decision Research online panel, a quote-recruited sample of U.S. adults	1, 4	Higher concern; Higher perceived US/global risk; Being Republican; Having higher education; Having less exposure knowledge;	Being older	Main outcome	Dec 08 – 21, 2014 (*n* = 815) Three weeks after second death from Ebola	8
Jung, Zhang, Nekmat 2020 [40]	Cross-sectional/Singapore	Zika	Third-person Effect	Singaporean women by employing Qualtrics panels, aged 20–49 years ol	1		Posting on social networking site	Mediator; Outcome: perform PHB	– (*n* = 510)	6
Karasneh et al. 2020 [41]	Cross-sectional/Unknown	COVID-19		Web-based questionnaire on pharmacists “nationwide”	1, 2, 3, 4	Living in a city; Working in hospital or community pharmacy; More frequent media watching; Certain sources of information; Having children		Main outcome	– (*n* = 486)	2
Lanciano et al. 2020 [42]	Cross-sectional/Italy	COVID-19		Web-survey via Google Forms, recruited through word of mouth; all Italians	1, 2, 3, 4	Being female; Having negative affective states; Having anxiety; Having loved ones living far from home; Having previous pathology	Having higher education; Having more perceived control	Main outcome	Apr 14 – Apr 19, 2020 (*n* = 980) Phase I Italian COVID lockdown	5
Li et al. 2021	Cross-sectional/China	COVID-19		Adults aged 18–85, online survey	1, 4	Stronger fate control (believing that fate is pre-destined)		Mediator; Outcome: ingroup bias	(*n* = 330) Initial outbreak in China	3
Liao et al. 2010 [43]	Cross-sectional/Hong Kong	H1N1	Theory of Reasoned Action/Planned Behaviour; Bandura’s Self Efficacy	Random-digit dialling, Kish grids; general adults	1, 4		Trust in interpersonal (informal) information	Mediator; Outcome: perform PHB	Jun 23–25, 2009 (*n* = 1,001) Two weeks after first community transmission	9
Liao et al. 2011 [44]	Cross-sectional/Hong Kong	H5N1, H1N1	Theory of Reasoned Action/Planned Behaviour; HBM; Social Learning	Anonymous random household telephone interviewing; general Hong Kong population	1, 2, 4	Trust in formal information	Trust in interpersonal (informal) information	Mediator; Outcome: trust in information; perform PHB	Dec 2005 – Mar 2006 (H5N1, *n* = 1,760) Southeast Asia epidemic peak May 13 – 15, 2009 (H1N1, *n* = 1,016) After global PHEIC, and first lab-confirmed case in Hong Kong	8
Lim et al. 2021 [45]	Longitudinal cohort/Singapore	COVID-19		Participants aged 16 and older (from an existing cohort study)	1, 4	Having more local cases; Increased trust in information from family; Increased trust in government communications	Being female	Outcome	Seven different survey times between Jan 24 – Apr 29, 2020 (*n* = 535)	6
Liu et al. 2020	Cross-sectional/China	COVID-19	SARF, HBM, PMT, Prevention Adaptation Process Model, Appraisal Tendency Framework	Residents in Chinese mainland through online survey platforms Tencent and Wenjuanxing	1, 4	Social networking site involvement (increase negative emotions)		Mediator; Outcome: perform PHB	No time specified (*n* = 711)	5
Lu, Schuldt 2018 [46]	Experiment/United States	Zika	The “nation as a body” metaphor	U.S. adults via Amazon’s Mechanial Turk	1, 4	Higher severity in messaging; More metaphoric (as opposed to literal) messaging		Mediator; Outcome: Perform PHB, Policy support	Feb 9 – Feb 15, 2017 (*n* = 354)	–
Malagón-Rojas, Parra, Mercado 2020 [47]	Cohort study/Colombia	COVID-19		Airport workers (male and female aged 18–60) at El Dorado International Airport	1, 3, 4	Having higher effect on life due to COVID-19; Concern about COVID-19 situation; Working at airport		Main outcome	June 1 – Sep 30, 2020 (followed every 21 days) (*n* = 212)	8
Mansilla et al. 2020 [48]	Cross-sectional/Spain	COVID-19		Residents of Spain, non-probability consecutive sampling (snowball)	1, 4	Direct experience with virus (family member or co-worker getting/dying from virus,); Working outside home; Having family member(s) or being someone who is (are) healthcare worker(s); Poor self-perception of health; Information from official media and scientific documents (compared to conventional media like press, radio, or TV); Being female		Main outcome	March 2020 (*n* = 16,372) During compulsory lockdown	6
Mohammadi et al. 2020 [49]	Cross-sectional/Iran	COVID-19		Iranian citizens from across country	1, 4	Being female; Having symptoms; More exposure to national media; Having positive relatives; More trust to foreign media; More trust to social media; More exposure to foreign media; More exposure to social media; More awareness of body sensations; Having feared consequence of having illness	Trust in national media; Trust to government	Mediator; Outcome: practising safety behaviours	Mar 5 – 22, 2020 (*n* = 1,881) First few weeks of Iranian outbreak	4
Nazione, Perrault, Pace 2021 [50]	Cross-sectional/United States	COVID-19	EPPM	Amazon MTurk participants from 47 states, aged 18–79	1, 4	More time spent on interpersonal conversations about COVID-19		Mediator; Outcome: Perform PHB	March 7, 2020 (*n* = 750)	8
Niño et al. 2021 [51]	Cross-sectional and panel study/United States	COVID-19		United States adults from the American Trends Panel and Diffusion of Fear survey	1, 4	Being a minority (Black-American, and Latino-American in initial wave, then Asian-American in later waves); Being female		Main outcome	March 10 – 16, 2020 (*n* = 8,914) Large growth in COVID-19 cases March 19 – 24, 2020 (*n* = 11,537) March 23 – 30, 2020 (*n* = 10,368)	9
Olagoke, Olagoke, Hughes 2020 [52]	Cross-sectional/United States	COVID-19		Residents aged 18 and over in the United States, through Qualtrics	1, 4	Increased news exposure		Mediator; Outcome: depression	Mar 25, 2020 (*n* = 501)	4
Pagnini et al. 2020 [53]	Cross-sectional/Italy	COVID-19		Italian residents aged 18 and over, recruited through snowball sampling on Facebook	1, 4	Having higher need for cognitive closure	Having higher emotional stability; Having higher mental health scores; Having higher levels of openness (creative and open to new experiences)	Main outcome	Feb 26 – Mar 04, 2020 (*n* = 2,886)	6
Park et al. 2021 [54]	Cross-sectional/ United States	COVID-19	HBM, TBP	U.S. residents aged 18 or older, recruited through Cloud Research MTurk Toolkit	1, 4		Having higher optimism bias	Mediator: Outcome: engagement in COVID-19 preventive behaviours/intentions	(*n* = 293)	6
Prati, Pietrantoni, Zani 2011 [55]	Cross-sectional/Italy	H1N1	Social-Cognitive Perception of Risk	Italians aged 18 years or older	1, 4	Exposure to media; More preparedness of institutions; Higher likelihood of infection; Higher levels of worry from family members and friends		Mediator; Outcome: perform PHB (mediated through affect)	Feb 16 – 19, 2010 (*n* = 1,010)	9
Rolison, Hanoch 2015 [56]	Cross-sectional/United States	Ebola		U..S residents recruited through Amazon MTurk	1, 2, 4		Having more knowledge of the disease	Main outcome	Nov 14 – 18, 2014	3
Rousseau et al. 2015 [21]	Mixed Methods/Multiple (France and Quebec)	H1N1		Info-Sante in Quebec (Ministry of Health call centre); Info-Grippe in France (Ministry of health phone line)	4	More media exposure		Main outcome	Mar 30 – 31, 2011 (*n* = 16 qualitative)	–
Rubaltelli et al. 2020 [20]	Repeated cross-sectional/Italy	COVID-19		Italian residents aged 18 and above	4	Later time/wave; More media exposure; Political orientation; Higher rate of cases		Predictor; Outcome: perform PHB	Feb 24 – 29, 2020 (*n* = 992) First outbreak wave and first deaths reported Mar 10 – 12, 2020 (*n* = 1,031) After country-wide lockdown	5
Rubsamen et al. 2015	Cross-sectional/ Germany	Ebola		Residents from four districts: Lower Saxony (Braunschweig, Salzgitter, Vechta, Wolfenbuttel), aged 15–69. Survey administered via Lime Survey	1, 3, 4	More use of media	Having higher knowledge score	Main outcome	November 2014 (*n* = 974)	5
Saleem et al. 2020	Cross-sectional/ Pakistan	COVID-19		Medical professionals from teaching and medical hospital in South Punjab	4		Having greater psychosocial strength (higher resilience, higher self-efficacy, more social support); Having higher self-control	Main outcome	March – April, 2020 (*n* = 284)	3
Samadipour, Ghardashi, Aghaei 2020 [57]	Cross-sectional/Iran	COVID-19		Social media users recruited through snowball sampling via WhatsApp and Telegram	Unspecified	Religious factors; Political factors; Cognitive factors; Social factors;	Emotional factors	Main outcome	Feb 25 – Mar 02, 2020 (*n* = 364) First week of outbreak in Iran	2
Savadori, Lauriola 2021 [58]	Cross-sectional/ Italy	COVID-19	Affect heuristic model; Cultural worldviews;	Italians aged 18 to 45, via Prolific platform	1, 4	Having a more individualistic worldview; Having a lower affective evaluation of the coronavirus		Mediator Outcome: adherence to protective behaviours	March 13 – May 4, 2020 (*n* = 572)	9
Schol et al. 2018 [59]	Cross-sectional/Netherlands	Ebola	HBM; PMT	General public and health care workers, recruited via a commercial research panel	3, 4	Being in healthcare industry (relative to general public); Being female; Higher knowledge level (in general public); Higher perceived susceptibility; Having children at home;	Having higher knowledge (as a health care worker)	Main outcome	Dec 12 – 14, 2014 (*n* = 526)	7
Seehuus et al. 2020 [60]	Cross-sectional/United States	COVID-19	PMT	Nationally representative sample through Qualtrics	1, 4	Race (white); Higher perceived impact on sleep due to pandemic		Main outcome	Apr 14 – 16, 2020 (*n* = 485)	4
Shauly, Stone, Gould 2020 [60]	Cross-sectional/ United States	COVID-19		Individuals aged 18 or older, from Amazon MTurk	4	Being older; Being female		Main outcome	March 24, 2020 (*n* = 969)	3
Winters et al. 2020 [61]	Repeated cross-sectional/Sierra Leone	Ebola		Knowledge, Attitudes, and Practice survey administered to participants recruited from housing census list	1, 4	Having higher education; Residing in Northern Province More use of new media and community source media; Having more knowledge on Ebola	Time (each next wave of the survey); Having more misconceptions about Ebola	Main outcome	Aug 2014 (*n* = 1,413) Three months into outbreak Oct 2014 (*n* = 2,086) Month before peak of outbreak Dec 2014 (*n* = 3,540) Month after peak of outbreak	7
Yang, Chu 2018 [62]	Experiment/United States	Ebola	Appraisal Tendency Framework	U.S. adults aged 18 and over from GfK Knowledge Networks	1	Being female; More conservative political ideology; Having more fear; Having more anxiety; Having more sadness; Having more anger; Having more disgust	Having higher household income;	Mediator; Outcome: support of institutional and personal mitigation measures combatting Ebola	Oct 21 – Nov 2, 2014 (*n* = 722)	–
Yang 2019 [63]	Experiment United States	Ebola	RISP	U.S. adults aged 18 and over from GfK KnowledgePanel	1	Being female	More psychological distance from Ebola	Mediator; Outcome: affective response; information seeking and processing	Oct 21 – Nov 4, 2014 (*n* = 1,046)	–
Yang, Xin 2020 [64]	Cross-sectional/China	COVID-19	PMT	Chinese citizens recruited from online survey platform	1, 4	Reliance on unofficial information sources; Reliance on official information sources	Reliance on official information sources	Mediator; Outcome: economic confidence	Feb 6 – 11, 2020 (*n* = 1,074) During spread of COVID-19	6
Ye, Lyu 2020 [65]	Cross-sectional/China	COVID-19	Trust, Confidence, and Cooperation Model; Salient Value Similarity; SARF	Chinese citizens	Unspecified	More trust in local government; More trust in local media (as opposed to central media)	Generalised trust	Mediator; Outcome: infection rate	2010 Chinese General Social Survey (risk perception data) (*n* = 11,783)	9
Zhong et al. 2020 [66]	Cross-sectional/China	COVID-19	PMT	Patients (diagnosed) in Fangcang hospitals	1, 2, 4		Having higher knowledge; Higher level of education	Main outcome	Feb 2020	2

^a^*ELM* Elaboration Likelihood Model, *EPPM* Extended Parallel Processing Model, *HBM* Health Belief Model, *PMT* Protection Motivation Theory, *PRISM* Planned Risk Information Seeking Model, *RISP* Risk Information Seeking and Processing, *SARF* Social Amplification of Risk

^b^1 = object of risk perception (who risk conceptualised for); 2 = risk against other disease; 3 = situational risk; 4 = risk on health-disease continuum; 5 = risk acceptance

^c^RP as an outcome depends on the Leppin-Aro concept (*e.g.* increased perceived risk vs. increased perceived severity vs. increased personal risk, etc.)

^d^NOS = Newcastle Ottawa Scale (adapted for cross-sectional studies). Full score out of 9, with 9 being highest

**Table 2:** Summary tables for epidemiology studies, literature reviews, and alternative studies assessing risk perception. Literature reviews assessing risk perception

Author and Year	Pandemic	Aim/Focus	Findings with regards to risk perception
Barrelet et al. 2013	H1N1	Description of lessons learned from H1N1 from a social sciences perspective; one section on risk and pandemic perception	Association with flu makes risk perception low; Tabloid reading and television made concern over H1N1 higher
Leppin, Aro 2009 [15]	SARS; H1N1	Review theory and models used in empirical studies for pandemic influenza and SARS	Most studies are not model-based, and under-theorised; Distilled out several “themes” regarding risk perception (used in the classification scheme above)
Majid et al. 2020 [18]	SARS; H1N1; MERS; Ebola; COVID-19	Understand relationship between knowledge, risk perception, and behaviour change	Higher levels of knowledge and risk perception promote uptake of hygiene and distancing behaviour; Higher knowledge may lead to higher perceived risk, and lower levels of fear; Misconception (usually from media) may decrease risk perception

**Table 3:** Summary tables for epidemiology studies, literature reviews, and alternative studies assessing risk perception. Alternative studies^b^ assessing risk perception

**Author and Year**	**Country**	**Pandemic**	**Theory**	**Method**^**a**^	**Leppin-Aro Concept**	**Factors potentially associated with RP**	**Where RP on pathway**	**Temporal/** **Time periods**
Chang 2012 [67]	Taiwan	H1N1	SARF; Framing theory	Assess Taiwan newspapers for alarm and coping frames Experiment of framing on perceived risk perception	2, 4	Having more alarming news frames (as opposed to coping frames) More frequent exposure to health-related news (especially by television, internet)	Outcome	No
Gozzi et al. 2020 [68]	Multiple	COVID-19		Mapping dissemination of information	–	Attention saturation (from media) can affect perceived risk	Outcome	No
Husnayain et al. 2020 [69]	South Korea	COVID-19		Use internet search data to understand risk perception (Google Trends; NAVER relative search volume)	–	Search queries by location indicate variation for risk perception	Outcome	No
Saxon et al. 2019	United States	Ebola	Extended Parallel Process Model	Analyse major news stories from five major US newspapers	4	Variable and ambiguous news reporting (not helpful for risk processing); Framing and variability of risk magnitude information; Having risk comparators	Outcome	No
Sell et al. 2018 [70]	United States	Zika	Issue Attention Theory; Framing Theory; SARF	Analyse 800 Zika-related news stories from 25 print and television sources	4	Message content from news can be risk-elevating or minimising	Outcome	No
Sesaigiri Raamkumar, Tan, Wee 2020	Multiple	COVID-19	HBM	Developing classifier for social media content along constructs of HBM, focusing on social-distancing as a topic	1, 4			No
Ye 2020	–	COVID-19		Developing a disease-behaviour-information transmission model	–	Information diffusion and disease awareness has impact on risk perception (measured as over-active nodes in a network)	Mediator; Outcome: epidemic numbers	No

^a^This section shows the alternative method used for risk perception research, and differs from the tables above in that no ‘population’ is explicitly mentioned

^b^‘Alternative studies’ refers to those using non-epidemiological methods for risk perception research

### Quality of studies

Out of the total 66 studies, only the cross-sectional studies (*n* = 52) were evaluated using the Newcastle Ottawa. Overall, the quality was moderate with an average of 5.4. Four studies had 4 stars, seven studies had 5 stars, nine had 6 stars, ten had 7 stars, five had 8 stars, and five had 9 stars, with none having a full score.

### Risk concept and associative factors

Included studies were also grouped by the Leppin and Aro classification on the concept of risk. This Classification categorises the concept of risk perception into five groups: (1) the object of risk perception; (2) comparison of risk against another disease risk; (3) situational risk; (4) the health-disease continuum; and (5) risk acceptance. No studies measured the concept of risk acceptance, and so this categorisation is excluded.

Included studies could, and often straddled two or more concepts of risk perception (*n* = 57, 91.9%), making categorisations non-mutually exclusive. Most studies were concerned about risk perception as part of the disease continuum (*n* = 55, 88.7%), followed by concern about who the risk was for (*n* = 47, 75.8%). Far fewer studies made a comparison about the PHEIC to another similar risk event (*n* = 9, 14.5%), and assessed situational risk (*n* = 6, 9.7%). The most common combination was conceptualising risk perception by identifying for whom the risk was, and to what the degree of risk was on the health-disease continuum (*i.e.*, the ‘who’ and ‘how severe’ of risk perception; or, groups 1 and 4 above). This is due to the fact that when asking participants about risk of infection or death, usually the object of risk is identified (*e.g.* “What is *your* risk of being *infected*?”). There are, however, few studies that ask about the risk on the disease-continuum without specifying the subject (*e.g.,* “What is the risk of *infection*?*”*, “What is the severity of COVID-19?”). Straddling two or more Leppin and Aro concepts makes summarising by individual risk concept difficult.

To summarise the risk factors on risk concept, the following is done. First, any studies with only one categorisation will be summarised. Second, double combinations of categories will be summarised, starting with the most frequently occurring pair of *who* the risk is for and the *severity*. This combination comprises the bulk of studies. Less common combinations are then discussed. Third, the studies using three-or-more conceptual categories are summarised. Finally, studies that can not be classified in these groups will be discussed as potential extensions onto the concept of risk perception.

### Single-category studies

Most single category studies focused on the disease-continuum concept of risk, and studied media and its contents as the determinant. Media was operationalised in several ways. Two studies found that the increased exposure of media would lead to an increase in risk perception [20, 21]. In a related study on media *attention*, Huynh found that the higher frequency use of social media was associated with a higher risk perception [37]. From these three studies, exposure and attention are complementary concepts: the former being a measure of the quantum of media, and the latter as user interest. More important than exposure and attention are media content. Dai et al*.* found that containing more positive risk information and detailed pandemic information was associated with higher risk perception [25]. These could be potential risk-elevating components, among many, as found by Sell et al*.* [70] In addition, Saxon et al*.* found that ambiguous news information was associated with the an increased risk perception [71].

Some studies, however, focused on different country contexts, and individual characteristics. For example, Huynh notes a geographical variation on increased risk perception, with those in central and southern Vietnam having higher risk perception than their northern counterparts [37]. Perhaps, this is due to the pandemic situation in those regions at the time, a finding corroborated by Rubaltelli et al*.* that a higher rate of cases would be associated with higher risk perception [20]. Shauly, Stone, and Gould find that generally older, female persons have higher risk perception [72]. Rubaltelli et al*.* suggests that political orientation may also be correlated with higher risk perception [20]. Other studies looked at psychological determinants of the individual. Saleem et al*.* finds that having greater psychosocial strength (higher resilience, self-efficacy, social support) is shown to be associated with *lower* risk perception of the virus, suggesting a robust social network helps reduce associated risk perception.

Two studies focused solely on the object of risk perception. These studies have no trends or overarching conclusions. One study corroborates the finding on gender in that being female is associated with higher risk perception [63]. The other study, however, finds that posting on a social networking site is associated with a lower perceived risk [40]. This finding is in contrast with the one earlier about media attention leading increased risk. The reasons for this can be because the underlying concept is not the same (media attention versus posting are two different actions); or, because of the different contexts (Jung et al*.* was conducted in Singapore, whereas the other studies are done in western countries).

### Double category studies

The bulk of studies were interested in who the risk was for, and to what degree on the health-disease spectrum it was. Determinants of risk perception can be separated into three broad categories: (1) individual-level determinants; (2) contextual determinants; and (3) media.

Individual-level determinants can be further separated into smaller groups: (1) emotion; (2) beliefs, trust, and perceptions; (3) individual immutable characteristics; (4) individual mutable characteristics; and (5) knowledge. On emotions, several studies are in accordance that having higher scores on anxiety and worry is associated with higher risk of infection [30, 73, 74]. More generally, persons that tend to be more reactionary or “deep” in negative emotions (having more sadness, more anger, more disgust, more depressive symptoms) are also associated with higher perceived risk of infection [30, 62]. This is corroborated in the other direction of emotion as a “protective” factor, whereby having higher positive affect [74] or higher emotional stability and mental health scores is associated with a lower risk perception [53].

On beliefs, trust, and perceptions, factors separate into whether it is of the self or towards the outside world. Of the self, those with higher belief in personal efficacy [29], more awareness of body sensations, more feared consequences of illness, [49], having higher need for cognitive closure [53], and national and global risk [39]) are all associated with higher perception of being infected. The finding on personal efficacy is contrasted with the finding by Han et al*.* that finds that higher self-efficacy is associated with a lower perception of risk [34]. Towards the outside world, some studies found that those with increased trust in science, medical practitioners, or government communications [29, 45] is associated with a higher risk perception. This is extended to those with higher trust in information in general, from foreign and social media [49] to information from the family [45]. However, the findings on trust are conflicting. One other study finds that trust in interpersonal (informal) information is associated with lower risk perception [43]. Another finds that trust in national media and government is associated with lower risk perception [29, 49]. These conflicting findings likely point to the importance of context when doing a risk perception study, and will be elaborated on later.

Individual immutable characteristics can be both a physical one (race, sex, age) or a psychological one (personalities). Findings in the physical category are very mixed. While some studies find that being female is associated with lower risk perception [45, 73], a majority of them find that being female is associated with higher risk perception [26, 28, 29, 35, 38, 48, 49, 51, 62]. Likewise, findings for age have no consensus. Some studies find that being older means higher risk perception [26, 33, 35], and some with lower [39, 74]. Two studies also show opposition in findings regarding ethnicity. One study finds that being a minority in the United States is associated with higher risk perception [51], whereas another finds that whites have higher risk perception [60]. Thus, the findings are inconclusive and likely depend on other factors such as sampling, context, and specific risk event. On the psychological front, some dispositions on personality may be associated with different risk perception, though findings are still dissonant. On a study looking at *optimism bias*, the phenomenon that one’s likelihood of experiencing good events is higher when compared to others, the finding is that those who have higher optimism bias usually also have lower risk perception [54]. Another study looks at the degree of openness an individual displays, finding that those with higher levels of openness towards new experience have lower risk perception [53].One study finds that those who appear more individualistic have a lower risk perception score [29], but a similar one finds the opposite relationship [58]. One finding from Farooq, Laato, and Islam find that users more pre-disposed to “cyberchondria” (excessive obsession of searching symptoms) will have higher risk perception [31]. Finally, one study also finds that those who are more altruistic or prosocial tend to have higher risk perception [29].

Another classification is mutable traits, which can include religious, educational, physical, domestic, economical, locational, and political factors. Religiously, those who are non-Muslim (in a majority Muslim country, Bangladesh) are associated with a lower risk perception [73]. Educationally, findings are diverging. Some studies find that having higher education is associated with higher risk perception [39, 61] while another finds the opposite association [26]. Duculan et al*.* looked specifically at the physical condition, studying risk perception in those with systemic rheumatic disease. They find that those having rheumatic condition, taking certain medications, having higher dependency on medications, and lower overall physical function is associated with higher perceived risk of infection [30]. This finding on medical fragility is echoed by a finding in Alschuler et al*.* that those with COVID-19 risk factors, such as pulmonary issues, also tend to have higher perceived risk of infection or death [74]. Domestically, one study finds that being unmarried is associated with higher risk perception [33], but living with children or in large households also shows a similar trend [35, 38]. For Zika in particular, those families who are planning to be pregnant also have higher risk perception [36]. Findings on income also dispute the direction of association, with some studies showing that higher income either means higher risk perception [33, 35] or lower [62]. Location-wise, only one study looks at the difference between urbanites and rural-dwellers, finding that those in urban environments have higher risk perception than rural counterparts [33]. In two studies in the United States, two studies are in accordance and find that Republican or conservative ideology is associated with higher risk perception [39, 62].

The last individual category is knowledge, specifically, knowledge either in a general sense or towards the specific PHEIC. In the general sense, two studies find that higher general knowledge is associated with higher risk perception [28, 29]. This directional finding is true also for the three studies that looked at PHEIC-specific knowledge [39, 61, 75].

The second broad category is about contextual determinants of risk perception, mostly referring “distance” from the pandemic. For example, those that have never undergone mandatory quarantine are more likely to have higher risk perception [73]. Those that: have been exposed to a confirmed suspect/case [28]; have more direct personal experience with the virus such as through knowing infected persons[29, 48]; are health care workers [33] or who have family members who are [48]; or live in areas that have more local cases [38, 45, 61] are all associated with a higher risk perception. Several factors are not just about pandemic proximity. For example, one study looked at how the preparedness of institutions in general may be associated with higher risk perceptions [55]. The same study also looked at how networks may also shape risk perception, with higher levels of worry form family members and friends associated to higher risk perception [55]. The last factor – time – is important but also understudied in the studies collected. One study finds that as time passes, there is an associated lower risk perception [61].

The third broad category is about media determinants of risk perception and can be split into three smaller categories: (1) exposure to media; (2) involvement with media; and (3) framing in media. This categorisation is similar to the one done earlier on the single-category studies. On exposure to media, those who received more information on the virus from family and friends [29]; increased exposure to social network sites [34]; have more exposure to national, foreign, and social media [32, 49, 52, 55] are all associated with higher risk perception. Exposure, while on the production side, likely elicits higher user involvement.

On involvement, there are several considerations. Those who have access to public and quality media [32, 48]; paid more attention to PHEIC information [34]; or increased interpersonal communications [34, 50, 76] all were associated with higher risk perception. In involvement, findings on how the quality of information intersects with engagement is nuanced. For example, one study finds that using television, community workers or free media websites was associated with higher risk perception. The same finding is noted in Winters et al*.* that finds using new media and community source media was associated in a similar direction. Yang and Xin corroborate this finding that reliance on unofficial information sources associates to higher risk perception [64]. However, based on these findings, one would assume that more trusted or official sources of information would run in the opposite direction. But, one study finds that receiving information from official media and scientific documents, when compared to conventional media like press, radio, or television, also is associated with higher risk perception. In addition, another study finds that those using WeChat (most popular social media messaging application in China) contacts as the main source of information actually is associated with lower risk perception [35]. Thus, there is no consensus on this association.

Lastly, only one study looked at framing in this double classification. Lu and Schuldt find that messages with higher severity, and those that are more metaphoric as opposed to literal, are associated with higher perceived risk [46].

While the *who* and *how severe* combination was the most common, there was one study which focused on situational risk and risk severity, as well as comparative risk and risk severity. The one study that focused on comparative risk and risk severity was done by Schol et al*.* In their study, their findings can largely be mapped onto the schema presented above. For example, Schol et al*.* found that there is an effect of belief on risk perception, such that those with higher perceived susceptibility associate with higher risk perception. Individual immutable factors such as being female show the same trend. Findings in knowledge are only partially congruent with the findings above. While higher knowledge levels are associated to higher risk perception, there is another finding in the opposite direction whereby those who have higher knowledge as a health care worker (the intersection) are associated with lower perceived risk compared to the public. Findings on context are largely congruent, indicating that those who work in the health care industry relative to the public are likely to have higher risk perception [59]. This specific focus of a situational risk (health care worker risk) distinguishes it from the study done by Harapan [33] in that the overall focus of the study is on situational risk, rather than a variable that was sampled by chance.

The other study focused on the combination of comparative risk and risk severity. This study took a different approach to traditional epidemiological studies, choosing to analyse newspaper frames for different diseases to understand how framing affects perceived risk perception. The findings are largely in accordance with those above, finding that having more alarming news frames, as opposed to coping frames, would shape risk perception (the direction is unclear). In addition, the findings on exposure are also the same. More frequent exposure to health-related news – especially through the television and internet – is also associated with risk perception [67].

### Triple and quadruple category studies

There were only two combinations of triple studies: ones combining the *who* and *severity* of risk with (1) a comparison risk event; or (2) a situational risk.

For the first combination, there were several studies that looked at factors that can be mapped onto the previous schema. On immutable individual trait, two studies found again that being female is associated with higher risk perception [23, 27]. This contributes to the findings in the previous section; however, not to the point of total consensus on the relationship between gender and risk perception. The same divergence is noted for knowledge. While one study found that having more knowledge of the disease is associated with higher risk perception [27], another found that it is actually negatively correlated with risk perception [56, 66]. Consensus, however, is reached on education, in that having more is associated with lower risk perception [27, 66]. One study looks at the beliefs, trust, and perception concept by focusing on the relationship between trust in information types and risk perception. This study finds that those who trust formal information have an associated higher risk perception, and lower risk perception for those who trust informal information [44].

Only two studies looked at the second combination. The first one found that more concern about the COVID-19 situation because of working at the airport was associated with higher risk perception [47]. This finding attests the importance of situation and context when doing risk perception research. The second study, despite assessing risk perception in scenarios, did not have any conclusive findings with regard to situation. Instead, they found that more use of media (media involvement or attention as mentioned earlier) was associated with higher risk perception [47]. Having higher knowledge score was associated with lower risk perception, again complicating the relationship between knowledge.

The last combination focused on all four categories of risk perception: who it was for, comparing it to another risk event, assessing situational risk, and risk severity. Both studies, however, collapsed the risk indicators for a summative metric, thereby also collapsing the concept of risk studied. For example, Karasneh et al*.* finds that working in a hospital or community pharmacy is associated with a higher risk perception, but also states that urban dwelling, more frequent media watching, accessing certain media, and having children are also all associated [41]. The other study lokes more at individual factors, finding that older persons, and those more knowledgeable about COVID-19 have higher perceived risk [22]. Also from this study is the finding that more preventive behaviours practice is associated with higher risk perception [22]. This point on the causality of risk perception [15] swill be mentioned later.

### No categories

Two studies had no clear, classifiable concept on risk perception. One study looked at various individual and contextual factors and their relationship to risk perception. These ‘factors’ are variable – including political, religious, cognitive, social and emotional – and vague. In addition, the measurement of the risk concept itself is not explicitly stated [57]. This conceptual weakness makes categorisation impossible. The other study focuses on the concept of trust as a predictor. It finds that more trust in local government and media (in the Chinese context) is associated with higher risk perception; however, ‘generalised trust’ is associated with lower risk perception. However, while this study is ranked highly by the Newcastle Ottoman Scale, the tool to identify the concept is unavailable, and thus the concept cannot be extrapolated.

Three studies did not fall clearly into any Leppin and Aro category. The first study, Gozzi et al*.*, looked at using different data sources, such as from Wikipedia, YouTube, and news channels, to study how attention saturation occurs [68]. While not mentioned explicitly, risk perception appears to be substituted by risk attention. The second study looked at a similar concept, using internet search data (*i.e.* attention) to proxy risk perception [69]. The last study used a behaviour-information transmission model to study risk perception. In Ye’s study, they use “overactive nodes in a network) to proxy risk perception, suggesting that disease awareness has an impact on risk perception [77]. All three of these studies seem to use attention to proxy risk perception. This is, however, not supported conceptually by other behavioural theories that conceptualise attention as a pre-processing stage before the processing – or risk perceiving – phase itself. For example, in the PADM, attention is considered as a pre-processing phase, for if no attention occurs, no consideration of the risk does [12]. Thus, using attention to proxy risk perception may not be conceptually correct, although it allows for novel methodological operationalisations on risk perception studies.

### Context – place, politics, time

Part of the difficulty in summarising risk perception studies is the varying contexts in which they occur. While nearly every country experienced COVID-19, other PHEICs like SARS, H1N1, Zika, and Ebola were much more localised with spreading potential. An example from this is the focus of U.S. studies to mostly be about H1N1, Zika, and Ebola (given the increased exposure of news on the infected case reaching U.S. soil); or, the fact that all China, Iran, and Italian studies are about COVID-19 (Italy and Iran were two notable outbreaks in March 2020 as the virus spread out of Asia). The degree to which countries experienced outbreaks is thus different, and furthermore, the cumulative experience likely shaped approaches towards later outbreaks.

In addition to different pandemic experiences, different political histories may also complicate the context in which pandemics occur, thereby influencing risk perception. This can happen both on an international or domestic level. For example, in a system with more political trust, risk perception for diseases may be much lower than compared to those with higher distrust. More comparative studies are needed to elucidate differences in political systems [78]. Domestically, differences in political belief and polarisation both account for a varied spectrum of risk perceptions on outbreaks, such as is found in the United States [20, 62, 79, 80]. The political context, coupled with pandemic experience, are also likely to interact and complicate what exactly “context” is.

Another difficult in summarising is because of time’s influence on risk perception. In the previous discussion, time was a strong predictor of decreasing risk perception, suggesting that timing of a study is important. This is also shown in the collected studies. Most studies chose to focus on a period in which some trigger related to the risk event occurred. A majority focussed on a pandemic situation-related moment (such as an outbreak, ongoing outbreak, reported death, or other trigger) [20, 28, 30, 32, 39, 43, 44, 49, 51, 57, 61, 81]; on when anti-epidemic measures were implemented or lifted [26, 32, 33, 35, 42, 48, 73]; or when certain declarations were made [37]. The choice on timing is important since risk perception is a process that is constantly reassessed. Leppin and Aro highlighted this issue, suggesting that more longitudinal studies are required to study the perception-behaviour correlation [15]. However, only a few studies implemented longitudinal studies, with most choosing to use cross-sectional designs. Continued surveying or monitoring of opinions such as through panel studies or regular opinion polling is thus important for consolidating findings on risk perception that are not confounded by time.

### Theoretical approaches

The Health Belief Model and Protection Motivation Theory were the two most common theories used (7 each). Four studies used them together to formulate their analysis. Since these theories are individual health behaviour models, it is unsurprising that the variables of focus were all on the individual level. Specifically, many looked at the relationship of gender [27, 38, 59], individual dispositions and decisions [31, 59, 82], age [75], ethnicity [60], trust [44], or other situational factors [59]. Several of them looked at the engagement with information [64] or social networking sites [83]. Another common theory was the Theory of Planned Behaviour, often paired as well with either the HBM or another theory.

Theories regarding the transfer and spreading of information also featured, and mostly focused on media factors. For example, one studying using the Protective Action Decision Model looked at how media framing would be associated with risk perception [25]. The Extended Parallel Processing Model, which looks at fear appeals, looked at how framing and variability of risk information links to risk perception [71] as well as how interpersonal communication is associated [50]. The Social Amplification of Risk framework was used by three research groups: Liu et al*.* to look at how social networking site involvement is associated with higher risk perception [83]; Chang to look at how news exposure, complemented by its either alarming or coping frames is associated with higher risk perception [67]; and Sell et al*.* to study how risk elevating, risk alarming messages may be related to risk perception.

The “other” theories category was a hodgepodge of theories depending on the researchers’ area of focus. For example, one study looked at how social networks and cognitions may shape risk, which would encompass variables from media to institutions to personal social networks [55]. Another study looks specifically at heuristic thinking and cultural worldviews – accounting for context – and finds that individualistic worldview thinking and heuristic evaluation of a PHEIC would be associated with higher risk of risk perception [58]. One final study uses the trust, confidence, and cooperation model to analyse how trust towards the government and media is associated with higher risk perception [65].

There was a statistical discrepancy on the association of studies using a theoretical basis to how they used risk perception as a variable (was it the outcome or mediator). Far fewer studies used theories when risk perception was the main outcome, as opposed to those in which risk perception was used as a mediator (X^2^ = 10.073, df = 1, *p* = 0.001). This is likely because if used as a mediator, risk perception must be conceptually linked to the outcome itself such that measuring of the constructs and the link is clearer. The most common outcome as performing public health behaviours (anti-epidemic behaviours), perhaps unsurprising since the studies all focused on PHEICs, and the most popular two theories – HBM and PMT – are individual health *behaviour* theories.

## Discussion

This review summarised several things. First, it consolidated factors that shaped risk perception, attempting to map them to different (and overlapping) concepts of risk perception. Second, it tried to understand how contexts may yield different findings. Last, it identified any theoretical approaches to risk perception studies. Most of the studies were cross-sectional, and a majority of studies were from North America (from the United States) and Asia (China). In addition, most studies chose to focus on two dimensions of risk perception, namely, who the risk was for and to what level it was on the disease-continuum (infection, death, etc*.*). Most studies did not have any theoretical basis for analytical approach, although those that did allowed the theory to drive the research questions and methods.

**Fig. 3:**
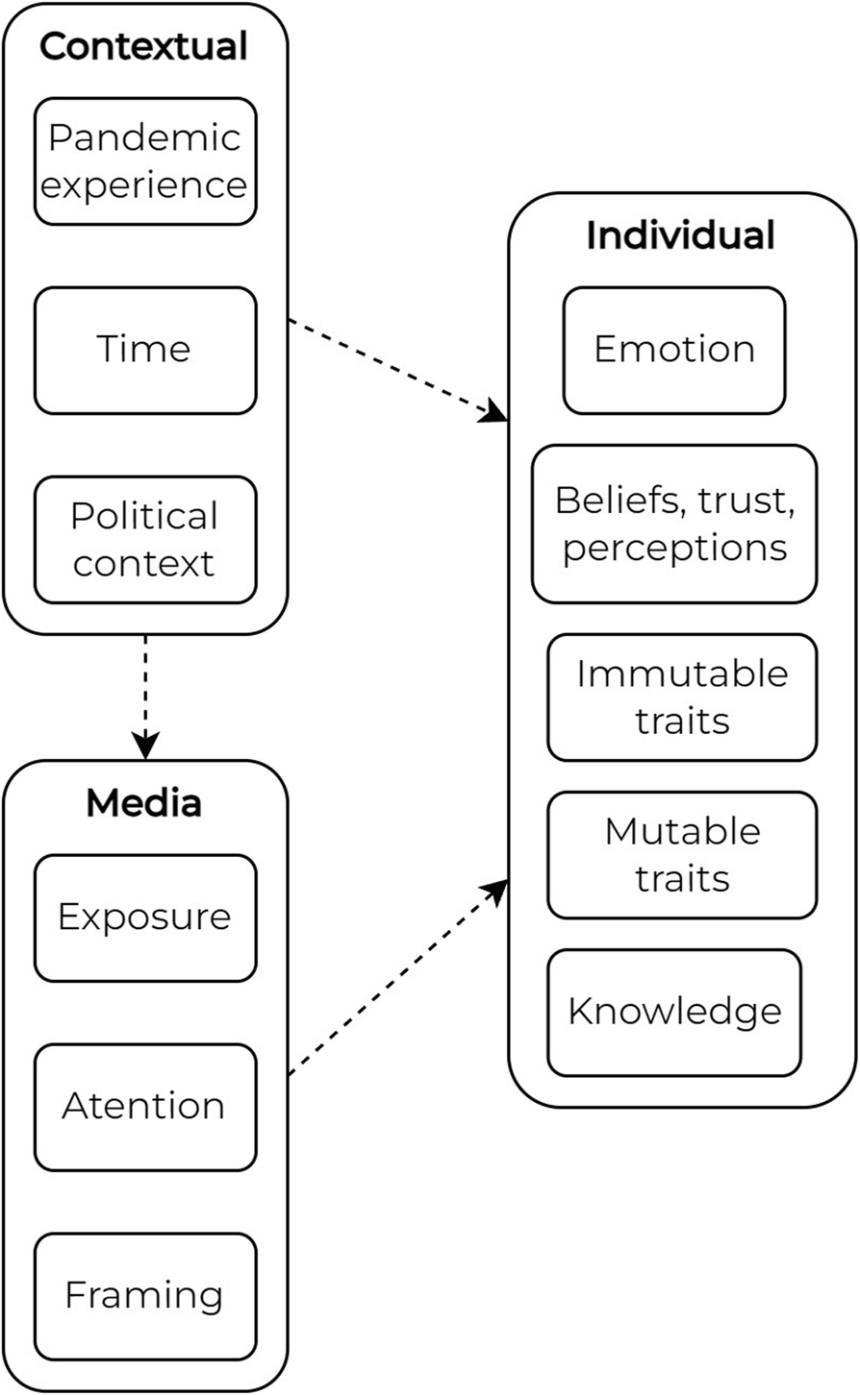
Classification schema for predictors of risk perception

The deduced factors are mapped out into Fig. 3. Overall, there are no clear patterns of the determinants of risk perception if classified by Leppin and Aro concept. While studies assessing only a single category would be most useful to deduce this, risk perception is often multi-conceptual in a study. This is shown by most studies that have two or more concepts included, as well as through some studies that choose to ask across many concepts and collapse the risk perception indicator to a single metric. However, multi-conceptualisation is particularly problematic since it the fundamental question of *what* aspect of risk perception is being measured is unclear, thereby complicating the consolidation of risk factors. We can assume, based on most studies, that they are concerned about the *risk of infection* for the *individual*. Most studies contain this combination, and even without explicit mention, gleaning the questionnaire also leads to this deduction. This is also indirectly inferred from popular health behaviour theories like HBM and PMT that attempt to measure *perceived severity* and *perceived susceptibility –* two concepts that capture who the risk is for, and the risk event of infection. Thus, while all results are associated with risk perception, caution must be taken as to interpreting *what* the factors are associating to. Also due to this unclarity, the determinants and their trends will be summarised across all studies.

Because the outcome often involves individual decision-making or health behaviours, individual determinants of risk perception feature heavily across all studies. Individual emotions are relatively congruent in their findings. Those who are more “negatively emotional” are associated with a higher risk perception, whereas those who are more “positively emotional” are associated with a lower risk perception. While not clear from individual studies, the summation of them makes a commentary about how *affect* – the encompassment of emotions – may be related to risk perception. The focus on affects, emotions, and mood is part of a larger movement from the 1960’s and 1970’s in health psychology on the importance of reactionary or emotional triggers when making decisions. For some theories such as EPPM, they specifically focus on how fear appeals in messaging can condition users to perceive the severity of a risk, and mobilise them to action [14]. Emotions are also closely related to beliefs, trust, and overall perceptions. Findings are inconsistent about how perceptiveness or awareness of the self, confidence in self-efficacy (in performing certain behaviours), or more fearful of consequences relates to risk perception. These, again, are important components for many health behaviour theories in Fig. 1. A growing body of literature in this space also is focusing on trust. While often overlooked, this determinant is important, although to what degree it does is debated with associations in both directions.

Perhaps the most conflicting set of predictors comes from the individual’s immutable and mutable characteristics. By nature of having so much variation, the relationship to risk perception is also complexified. There are no clear findings on race, sex, age, religion, education, or income. Certain personality dispositions such as openness, individualism, or pro-socialness also have little to no evidence of support. The only clear relationship from these studies is the importance of knowledge, both in a general sense and towards the PHEIC. Those with higher knowledge are associated with a higher risk perception across all studies looking at it.

This lack of consensus can be due to several things. First, as mentioned it before, it can be because of the vagueness of what risk perception is measuring: if the outcome is not the same, then the determinants and their relationships will not be. So, for future risk perception studies, absolute conceptual clarity on the risk perception concept should be defined. Second, and equally important, is the importance and need to account for the context where the study takes place. There are likely macroscopic forces of context that shape the individual experience, especially in the realms of beliefs and trust towards institutions, as well as knowledge or individual mutable factors. Thus, in Fig. 3, a dashed line is drawn to the individual to indicate how these two factors are related.

Context refers to several things. First is the experience with the pandemic, which can be both at a national (or jurisdictional) level, as well as an individual level. Using COVID-19 as an example, for east Asian states, the pandemic began in early January 2020, where border closures, mask wearing, and early anti-epidemic measures were employed soon after the first cases were identified in corresponding jurisdictions. These systems and institutions were largely developed as a part of earlier scares of SARS and H1N1. Thus, the pandemic experience was a lot sooner, and a lot closer. In addition, these states collectively were overall more compliant with anti-epidemic measures, both at the beginning and throughout the pandemic (at the time of writing in December 2022, Hong Kong still mandated outdoor mask-wearing at all times). At the individual level, those who have already experienced infection, or were “closer” to the virus by being a high-risk working group (doctors, nurses, airport staff) or exposed to the virus will have different risk perceptions than the public. The second is time. Most studies in this review chose specific times that were informed by the pandemic experience. For example, specifying a time during a major outbreak, an announced lockdown, or a declaration of emergency all serve as ideal periods of how triggers or signals shape risk perception. Since risk perception constantly fluctuates, capturing the time aspect is important. While most studies capture this aspect this through a snapshot technique, as exemplified by most cross-sectional studies, they may not be as informative as longitudinal or panel studies that look at risk perception. The reason for this is because of the predicament of disentangling the timing link between risk perception and behaviour (most studies tend to use risk perception as a mediator to link to behaviour). Leppin and Aro discussed this briefly in their study, referring to the correlational link between risk perception and behaviour only an *accuracy hypothesis* [15]*,* and not one of *behaviour motivation* or *risk appraisal.* The failure to capture this component of time often will lead to misinterpretation of findings. More studies should be designed longitudinally to understand this relationship. The last is political context. This theme is not often explored on an international comparative level. and more room to understand how political economy shapes risk perception could better illustrate this factor.

Another large factor that is also shaped by context is media (arrow drawn in Fig. 3). Most studies agree that an increased exposure – the quantum of media – is associated with higher risk perception. Theories such as the Social Amplification of Risk Framework by Kasperson [84] would support this. As more outlets or “information stations” transmit the risk event, this increased exposure inadvertently promulgates risk messages, thus amplifying them. However, exposure is not enough. Audience engagement or involvement with media information is also important. The studies in this review find that the types of media that users engage with are associated with risk perception; however, there is no consensus on what types may be associated with higher or lower risk perception. Given the complicated media landscape with the advent of social media, this link is difficult to disentangle since what information an individual is exposed to or processes is very complex. In addition, when individuals become amplification stations on social media platforms, some potentially with wide reach, their message reach may outstrip those of traditional sources. This affects the issue-attention cycle [85, 86] of outbreaks, drawing attention as to what is deemed a salient issue in the public sphere. In addition, this could also have implications for when certain policy windows open as part of the larger policy cycle. For risk perception, depending on what messages are being sent through framing tactics, there are potentially large implications. From the few studies looking at framing, those with more severe or metaphoric messaging are associated with higher severity [46, 70], and so looking into what messages are being transmitted is an important endeavour. A growing body of literature has looked at the effects of misinformation on shaping behaviours during an outbreak, most notably for vaccination.

One major limitation of this review is the right-censor date of end-2020, when the COVID-19 pandemic was still unfolding. Since then, many more studies on risk perception of COVID-19 have been completed, sampled at various times, further complexifying the results. While these studies warrant inclusion, whether they contribute to any consensus on predictors of risk perception during PHEICs is unknown.

Still, with the lack of consensus across all studies, there should be a more consolidated approach towards risk perception studies. This is through a grounding of the studies in theory. From this review, we see that anchoring the studies to the theory guides the questioning, measurement of variables, and interpretation of result into a clearer, a more coherent body of work. In addition, conceptual clarification on what aspect of risk perception is begin measured is important. This review suggests that the most common focus is on individual infection risk. Furthermore, a pure focus on just risk perception is not the end goal. Asking whether a factor is related to higher or lower risk perception misses the point of what these studies ultimately strive to answer – that is, how it shapes behaviour. More longitudinal studies are better at disentangling this relationship due to the sensitivity of time of the perception-behaviour interface. And, finally, considering all of these in a specific context, with its own pandemic trajectory, history, and culture is also incredibly important. Risk perception, while an individual trait, is shaped by a multitude of larger, macroscopic factors that shape how individuals think and act, especially during a crisis.

## Data Availability

All data generated or analysed during this study are included in this published article’s tables.
